# Improving Chromatin-Interaction Prediction Using Single-Cell Open-Chromatin Profiles and Making Insight Into the *Cis*-Regulatory Landscape of the Human Brain

**DOI:** 10.3389/fgene.2021.738194

**Published:** 2021-10-08

**Authors:** Neetesh Pandey, Omkar Chandra, Shreya Mishra, Vibhor Kumar

**Affiliations:** Department of Computational Biology, Indraprastha Institute of Information Technology, New Delhi, India

**Keywords:** chromatin-interaction, single-cell epigenome, single-cell ATAC-seq, GWAS target, long-range, brain *cis*-interaction

## Abstract

Single-cell open-chromatin profiles have the potential to reveal the pattern of chromatin-interaction in a cell type. However, currently available *cis*-regulatory network prediction methods using single-cell open-chromatin profiles focus more on local chromatin interactions despite the fact that long-range interactions among genomic sites play a significant role in gene regulation. Here, we propose a method that predicts both short and long-range interactions among genomic sites using single-cell open chromatin profiles. Our method, termed as single-cell epigenome based chromatin-interaction analysis (scEChIA) exploits signal imputation and refined L1 regularization. For a few single-cell open-chromatin profiles, scEChIA outperformed other tools even in terms of accuracy of prediction. Using scEChIA, we predicted almost 0.7 million interactions among genomic sites across seven cell types in the human brain. Further analysis revealed cell type for connection between genes and expression quantitative trait locus (eQTL) in the human brain and making insight about target genes of human-accelerated-elements and disease-associated mutations. Our analysis enabled by scEChIA also hints about the possible action of a few transcription factors (TFs), especially through long-range interaction in brain endothelial cells.

## Introduction

Spatial interactions between different genomic loci are required for multiple regulatory functions (de Wit and de Laat, 2012). Many groups have profiled chromatin-interaction in multiple cell types using different experimental high-throughput methods to study such complex patterns in chromatin architecture and gene regulation. The experimental methods based on chromosome conformation capture (3C) are more focused on local genomic loci (Dekker et al., 2002). The Chromatin-interaction Analysis by Paired-End Tag Sequencing (ChIA-PET) method captures distal interactions, but it is limited to only binding sites of the protein of interest (Tang et al., 2015). The high-throughput chromosome conformation capture (HiC) assay provides a genome-wide chromatin-interaction profile but requires deep-sequencing to achieve high resolution (de Wit and de Laat, 2012).

Several groups have recently attempted to predict chromatin interactions using linear one dimensional genetic and epigenetic information (Li et al., 2019). Most of the tools proposed for predicting interaction depend on the use of epigenetic information from bulk samples, often consisting of multiple cell types (Whalen et al., 2016). Simultaneous availability of many epigenome profiles is currently possible for only a few cell types. Hence predicting cell-type-specific chromatin-interaction is not trivial for many cell types. On the other hand, if we exploit heterogeneity in the activity of genomic sites in single-cells, we could predict chromatin interactions in a cell type. Especially for understanding regulatory mechanisms in minor cell types in heterogeneous clinical samples for personalized therapy, single-cell epigenome profiles can provide the landscape of genomic sites’ activity and the prediction of chromatin-interaction. With experimental assays like 3C and HiC and computational methods using bulk epigenome profiles, it would not be trivial to profile chromatin-interaction maps for multiple cell types for heterogeneous clinical samples from patients on a regular basis. Recently, Pliner et al. (2018) proposed a method called Cicero to predict local chromatin-interaction using single-cell Assay for Transposase-Accessible Chromatin using sequencing (scATAC-seq) profile. However, Cicero is designed for predicting interactions among genomic sites, which are within 500 kbp (kilobase pairs) of each other. Another method called jointly reconstruct *cis*-regulatory interaction maps (JRIM) (Dong and Zhang, 2020) uses open chromatin profiles of multiple cell types to infer reliable chromatin interactions; hence it is of less use for prediction for a single cell type. JRIM is also designed to predict chromatin interactions within 500 kbp window. However, it has been shown before that mutation identified by genome-wide association studies (GWAS) could be influencing genes lying more than 500 kbp away. The median size of the topologically associated domain (TAD) in mouse cells have been reported to 880 kbp (Dixon et al., 2012). Previously Novo et al. (2018) highlighted the significant role of long-range interactions (>800 kbp) among promoters and super-enhancers in poising and activation of embryonic stem cells (ESCs). Similarly, other studies have also highlighted the importance of long-range interaction for understanding gene-regulatory patterns and related epigenetic profiles (Ling and Hoffman, 2007). Hence, predicting long-range (distal) chromatin interactions using a single-cell epigenome profile is an important open problem of high utility. Here we developed a method called as single-cell Epigenome based chromatin-interaction Analysis (scEChIA), which can predict interactions among distal sites with high accuracy using single-cell open-chromatin profiles. We have further shown its utility in the prediction of chromatin interactions in brain cells for making useful insights.

## Materials and Methods

### Pre-processing of Data

Our tool first divides the genome into bins of the required size. By default, it uses a bin of size 25 kbp. For a read-count matrix, it merges the peaks lying within the same bin. For merging two peaks, it adds their read-counts. After merging the peaks, it takes log transformation of the new read-count matrix as


(1) $${\bar{x}}_{i\,j}=l\,o\,g\,\left(x_{i\,j}+1\right)$$

### Gaussian Graphical Model With an Improved Penalizing Parameter to Embed Previous Knowledge

In the read-count matrix of the single-cell open-chromatin profile, the number of peaks is often more than cells. Hence the estimation of a matrix with covariances of peak activity is not trivial. For such problems, Gaussian graphical model, such as graphical-Lasso (Friedman et al., 2008) method helps in estimating regularized covariance matrix and its inverse. The inverse of the covariance matrix can be used to calculate partial correlations. Here partial correlation provides the degree of co-accessibility between peaks after removing the effect of confounding factors due to other peaks. Graphical Lasso is used for detecting such direct association among variables. The penalty term in Graphical lasso causes shrinkage of partial correlations between peaks pairs (Friedman et al., 2008), when the strength of their association is low. The Graphical Lasso method tries to maximize:


(2) $$l\,o\,g\,d\,e\,t\,\Theta -t\,r\,\left(U\,\Theta \right)-\rho \,{∥\Theta ∥}_{1}$$

where Θ is the inverse covariance matrix and *U* is the covariance matrix, and ρ is the penalty term for L1 norm based regularization. The penalty term can be a matrix consisting of different ρ values for each pair of variables (peaks). Our method uses a penalty matrix which is designed differently based on the knowledge of pre-existing chromatin-interaction profile. The elements of the penalty matrix are calculated as


(3) $$\rho_{i\,j}=\frac{\delta }{h_{i\,j}+\varepsilon }$$

where [*h*_*ij*_] is the average enrichment level of chromatin-interaction between genomic bins [*i and j*] estimated using published HiC profile of multiple cell-types. The term ε stands for a pseudo-count to stop the inflation of penalty terms in case no chromatin-interaction is found in the available HiC profiles. Whereas [δ] is a constant which can be adjusted to increase or decrease the number of predicted interactions at the cost of accuracy. The design of our method is also meant to handle the following cases:

1. When two interacting sites have high activity in all cell types, and the drop-out in their read-counts is due to stochasticity and lower sensitivity during scATAC-seq profiling, then the covariance between them might be under-estimated. However, if their interaction is present in all cell types, giving a lower penalty or higher prior value would help retrieve that information.
2. If the noise level in read-counts of single-cell open chromatin profile is high, then a prior guess about the background could lead to an improved prediction of interaction.
3. If two sites have cell-type-specific interactions and have a decent covariance value, it could still be retrieved as the penalty is not exponentially high. Here decent means higher value in comparison to most of the other elements in the covariance matrix.

Hence prior knowledge (or guess of penalty matrix) is a crucial step. In order to further improve the prediction, scEChIA uses matrix-factorization to reduce noise in the read-count. The matrix-factorization used by scEChIA is described below.

### Matrix Factorization to Improve Co-occurrence Estimation

Matrix factorization is a method for low-rank matrix completion problems. An observed read-count matrix Y, where columns represent peaks, and each row represents a cell, can be called a sampled version of true ideal matrix *X* of the same dimension (m × n). Such that


(4) Y=A⁢(X)

Here *A* is an operator matrix and has 0′s where the elements of X is missing in Y and 1′s where it is present. However, if X is known to have a rank *r* (<*m, n*), X can be written as a product of two matrices *U*_*m* × *r*_ and *V*_*r* × *n*_. Therefore, Y can be written as


(5) Y=A⁢(X)=A⁢(U⁢V)

In order to recover X we try to find matrix U and V by minimizing the Ferbius norm of following cost function


(6) $$\begin{array}{c} m\,i\,n\\ u,v \end{array}\,||Y-A\,\left(U\,V\right)||\,\begin{array}{c} 2\\ F \end{array}$$

In order to optimize such bilinear problems, we use Majorization-Minimization (MM) (Sun et al., 2017). For MM based optimization, a surrogate function that majorizes the objective function is chosen. The surrogate function is then minimized until a local optimum is achieved. To minimize our cost function given in Eq. 6 the majorization step is implemented such that we optimize


(7) $$\begin{array}{c} m\,i\,n\\ u,v \end{array}\,||B-A\,\left(U\,V\right)||\,\begin{array}{c} 2\\ F \end{array}$$

where [$B_{k+1}=X_{k}+\frac{1}{a}\,A^{T}\,\left(Y-A\,\left(X_{k}\right)\right)$] at each iteration k. Here [*a*] has scalar value and [*X*_*k*_] is the matrix calculated as iteration k as [*X*_*k*_ = *U*_*k*_*V*_*k*_]. Here the matrices U and V are updated in an alternative manner such that when U is updated when V is considered to remain unchanged. Then V is updated while keeping U as fixed.


(8) $$U_{k}=||B-U_{k-1}\,V_{k-1}||\,\begin{array}{c} 2\\ F \end{array}$$

(9) $$V_{k}=||B-U_{k}\,V_{k-1}||\,\begin{array}{c} 2\\ F \end{array}$$

We keep a non-negativity constraint on X such that after every iteration, we truncate the element with a negative value in [*X*_*k*_] to zero. We initialize factor V as a matrix with r right singular vector of X after singular value decomposition (SVD) of X. SVD is a generalization of eigenvalue decomposition for rectangular matrix such that matrix X (size: m × n) can be represented as


(10) X=L⁢∑R

where [∑]is rectangular diagonal matrix of size m × n and matrix and [*L*] is m × m matrix and [*R*] is n × n matrix. Here choose r vectors from the right matrix [*R*] to make initial guess of matrix [*V*].

### Evaluation of the Accuracy of Prediction of Chromatin Interaction

For evaluation of the accuracy of prediction of chromatin-interaction, we used published HiC profile in respective cell-type. We first extracted chromatin interaction in text format at 25 kbp resolution from .hic file using juicer-tool (Durand et al., 2016). The three column output from juicer-tool was converted to seven column format. For the evaluation purpose, a threshold was used to choose only top enriched chromatin interactions from HiC profile. We used two ways to choose top enriched chromatin interactions from HiC profile. In the first way, we chose top 60,000 chromatin-interaction in every chromosome from .hic file. According to the second way, the number of selected chromatin interactions from HiC profile was proportional to the size of chromosomes such that the highest number of interactions was 60,000 for the longest chromosome. PGLtool was used to intersect the predicted chromatin-interaction with HiC based output (Greenwald et al., 2017).

### Parameters Used for Predicting Chromatin-Interaction

Cicero provides a few functions for pre-processing, such as make_atac_cds, aggregate_nearby_peaks, detectGenes, estimateSizeFactors, reduceDimension, make_cicero_cds, estimate_distance_parameter, generate_cicero_models, assemble_connections. The parameter for function aggregate_nearby_peaks was distance = 25,000 and for function reduceDimension, max_components = 2, num_dim = 3, reduction_method = tSNE, perplexity = 5. For subset function the Hg19 genome version was used and estimate_distance_parameter function was given the window size of 500,000. The rest of the functions were used as default parameters.

For scEChIA, we used functions with different rho options, such as rhomatAvg and Interaction_Prediction_1. Using the function rhomatAvg, we calculate the average of two different HiC file. The bin size was chosen to be 25 kbp and provided chrNo and patternf according to the chromosome number. The function Interaction_Prediction_1 was used to predict chromatin interaction using background information as an average HiC matrix and other variables like chrinfo, data, rhomatrix, chrNo, startCell, endCell, and chromSize. Function ucscTrack was used to make a UCSC Track file, and that was based on predicted interaction. For constant_rho we used a function Interaction_Prediction_2 that was based on constant rho 0.01.

### Data Sources

The scATAC-seq profile for K562, H1ESC, and GM12878 cells is available by Buenrostro et al. (2015) with GEO ID: GSE65360. The single-cell open chromatin and expression profiles of brain cells published by Lake et al. (2018) and used here are available in GEO database (GEO ID: GSE97942). The single-cell open-chromatin profile for cardiomyocytes (Domcke et al., 2020) is available with GEO ID:GSE149683. The chromatin interaction profile determined by HiC and used here for evaluation are available at 4D_nucleome database^1^ with IDs: Astrocytes- 4DNFITPO1WTY, cardiomyocytes- 4DNFIN39NO4O, GM12878- 4DNFIPAI8XB5, hESC- 4DNFIOX3BGNE, K562- 4DNFI8Y9SRP2. For K562, H1ESC, and GM12878 cells HiC data from Rao et al. (2014) (GEO ID: GSE63525) was also used to confirm the results.

## Results

Tang et al. (2015) have shown that in spite of many cell-type-specific interactions, multiple chromatin interactions show high similarity across different cell-type. It is known that CCCTC-binding factor (CTCF) mediated chromatin-interaction and looping are mostly conserved and have a major impact on chromatin architecture. Similarly, many short tandem repeats define boundaries of the TADs, which tend to be conserved across different cell types (Sun et al., 2018). Our computational approach is based on the well-known property of conservation of DNA looping and chromatin conformation. Hence, to avoid limitations faced by previous methods, we used existing knowledge of chromatin-interactions in multiple cell-types as a constraint factor while estimating the Gaussian *graphical* model, using L1 regularization to predict chromatin-interaction using a single-cell open-chromatin profile. For this purpose, we use the average value of enrichment of known chromatin-interactions in multiple cell-types to calculate L1 regularization (ρ) parameter. In addition to using sensitive L1 normalization, scEChIA uses its inbuilt function for matrix factorization to reduce noise in the read-count matrix to further improve the accuracy of prediction of chromatin-interaction (see section “Materials and Methods”).

### Single-Cell Epigenome Based Chromatin-Interaction Analysis Improves Sensitivity for Predicting Distal Interactions With High Accuracy

**FIGURE 1:**
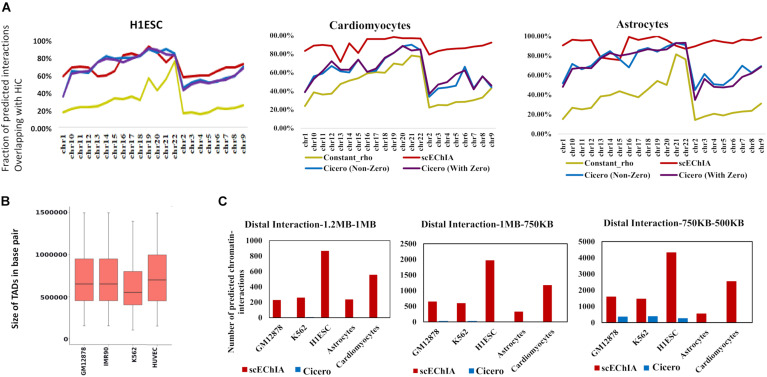
Evaluation of accuracy and sensitivity of chromatin-interaction prediction. **(A)** Accuracy of prediction of chromatin-interaction using single-cell open chromatin profile is shown for three methods: Glasso used with constant regularization parameter (constant_rho), Cicero, and scEChIA. For Cicero, two results are shown; one without taking predicted interaction with zero scores and the other comprising all interactions. Accuracy was measured as the fraction of predicted chromatin interactions, which overlapped with enriched interactions (top 60,000 in each chromosome) in the HiC profile of the respective cell (see Supplementary Material). **(B)** The sizes of topologically associated domains (TADs) in different types of human cells. The TAD boundaries are made available by Liu et al. (2019) at (http://dna.cs.miami.edu/TADKB/). The median TAD size is more than 500 kbp. **(C)** The sensitivity of prediction of distal chromatin-interaction is shown here for different ranges of the distance between interacting genomic loci. scEChIA breaks the barrier of fixed-distance criteria and is 1,000 times more sensitive in predicting long-range chromatin-interactions (>500 kbp).

We compared our method’s accuracy and sensitivity with the famous method Cicero (6). For this purpose, we used scATAC-seq data-set of K562, GM12878, and H1ESC published by Buenrostro et al. (2015) and single-cell open-chromatin profile of astrocytes (Lake et al., 2018) and cardiomyocytes (Domcke et al., 2020). We calculated the regularization parameter ρ in graphical Lasso (Glasso) model using the average of known chromatin-interaction in other cell-types for predicting chromatin-interaction for a cell-type. For example, for predicting chromatin-interaction in K562 cells we used prior (or regularization parameter ρ) estimated using the average of HiC profile of GM12878 and H1ESC cells (Rao et al., 2014). For GM12878 cells we used scATAC-seq profile published by Buenrostro et al. (2015) and the average of HiC profile of K562 and H1ESC to calculate the regularization parameter. We performed an evaluation using HiC based enriched chromatin-interaction profile of relevant cell types (see section “Materials and Methods”). We found that using a single regularization parameter (constant ρ) value with Glasso did not provide comparable accuracy in predicting chromatin-interaction (Figure 1A). Due to the refined regularization matrix, scEChIA had better performance than Cicero for all chromosomes for 2 out 5 cell lines used for evaluation (cardiomyocytes and astrocytes) (see Figure 1A and Supplementary Figure 1). Whereas for the other three types scEChIA and Cicero had similar performance. We confirmed our results with two types of thresholding criteria for choosing significant chromatin interaction using HiC data. As shown in Figure 1A and Supplementary Figure 1, we first used the top 60,000 chromatin interactions in HiC profile of every chromosome as a positive set for evaluation of predicted interactions. Further, we also confirmed our finding when the number of HiC based interactions varied according to the size of the chromosome (Supplementary Figure 2A and Supplementary Table 1). Thus, scEChIA also tend to outperform other methods on some data-sets of the single-cell open-chromatin profile in terms of predicting correct interactions.

It has been shown that the median size of the TAD in mouse cells is approximately 880 kbp (Dixon et al., 2012). Since we used the scATAC-seq profile of human cells, it was important to measure size of TADs in human cells. Therefore, we used published TAD boundaries at TADKB database and found that the median TAD size in human cells is also more than 500 kbp (Figure 1B). having confirmed the large sizes of TADs, we further counted the number of long-range interactions predicted by different methods. As expected, scEChIA predicts a substantially higher number (almost 100 times) of long-range interactions with a gap of more than 500 kbp among interacting sites (Figure 1C) without losing sensitivity for short-range chromatin-contacts (Supplementary Figure 2B). We confirmed the substantially higher sensitivity for scEChIA for long-range interaction (>500 kbp) for five cell types (GM12878, K562, H1ESC, astrocytes, and cardiomyocytes). Overall, the estimate of large TAD sizes, highlights the importance of detecting long-range interaction to capture interTAD interactions, which could be made feasible using scATAC-seq by scEChIA.

### Evaluating Cell-Type Specificity of Predicted Interactions and Their Effect on Gene Expression

Even though we could predict both short and long-range interactions, a doubt remained about their relevance with gene expression and cell-type specificity. Our further analysis revealed that genes with more number of predicted chromatin interactions had higher expression in comparison to genes with low connectivity (Supplementary Figure 3). Thus, predicted interactions by scEChIA tend to be coherent with gene-expression profiles. We also tried highlighting predicted cell-type-specific interactions and their consequential effect on gene expression. Comparing predicted chromatin interactions in three cell types (K562, GM12878, and H1ESC) we found many genes with a higher relative number of chromatin-interaction at their promoters. Such results show that the number of predicted chromatin interactions at promoters of different genes varies according to cell types.

**FIGURE 2:**
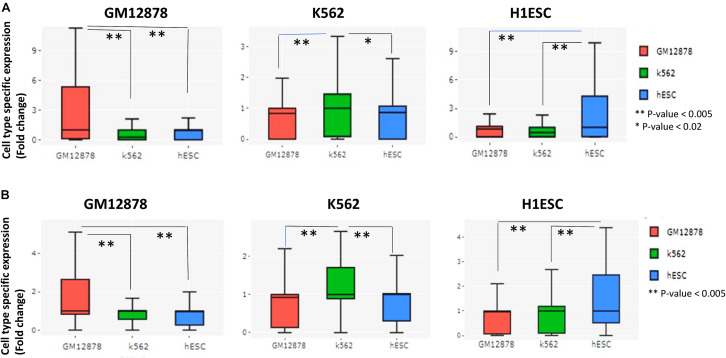
Evaluation of prediction of cell-type-specific *cis*-regulation by scEChIA. **(A)** The cell-type-specific expression (fold-change above-median across different cell-types) of top 200 genes with the highest relative interactions in the respective cell-type of the figure panel. Such as, for the panel with label GM12878, top 200 genes with the highest relative number of chromatin-interaction in GM12878 cells (compared to K562 and hESC) were chosen and their expression values in three cell types (GM12878, K562, and hESC) are shown as box-plot. These results indicate that scEChIA can predict *cis*-regulatory interactions, which are associated with cell-type-specific expression. **(B)** The cell-type-specific expression of top 200 genes with the highest relative long-range interactions (>500 kbp) in the respective cell-type of the figure panel. Many long-range interactions (>500 kbp) predicted by scEChIA are also associated with cell-type-specific expression. *Significant, **Most Significant. *p*-values were calculated using Wilcoxon rank sum test.

Moreover, genes with a higher relative number of predicted chromatin-interaction had higher expression in respective cell-type whose scATAC-seq profile was used for prediction (Figure 2A). Thus, scEChIA also predicts cell-type-specific interactions, which regulate the specificity of the activity of genes according to cell types. Further, we repeated the same procedure using only predicted long-range interactions. Again we found that genes with a higher relative number of predicted long-range chromatin-interaction had higher expression in respective cell types (Figure 2B). These results confirm that scEChIA also predicts cell-type-specific long-range chromatin interactions, which influence the specificity of the activity of genes.

### The Chromatin-Interaction Landscape of the Human Brain

Recently, Lake et al. (2018) published single-cell RNA-seq and single-cell open-chromatin profiles of cells derived from the adult human brain. For profiling single-cell open chromatin patterns, Lake et al. (2018) used single-cell transposome hypersensitive-site sequencing (scTHS-seq), to achieve higher sensitivity than ATAC-seq. The high accuracy in predicting chromatin interactions astrocytes using scTHS-seq profile by scEChIA (Figure 1A) also hints about higher chances of accurate prediction for other six *brain* cell types. Thus, we used scEChIA to predict chromatin-interaction in other six brain cell-types using scTHS-seq profile published by Lake et al. (2018). The cell types for which we predicted chromatin-interaction are inhibitory neurons, excitatory neurons, astrocytes, oligodendrocytes, oligodendrocyte precursor, microglia, and endothelial cells. The number of predicted chromatin-interaction in different cell types ranged from 188857 in Microglia to 25838 in Oligodendrocytes (total ∼0.7 million interactions) (see Supplementary Table 2).

**FIGURE 3:**
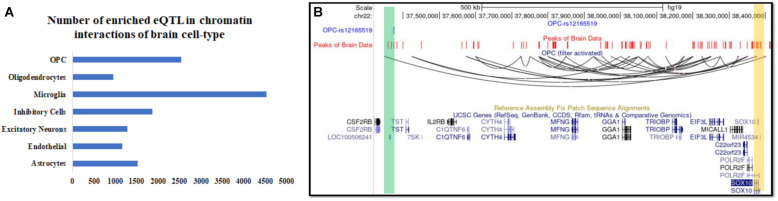
Inference about the cell-type of action and associated known target genes for expression QTL (eQTL) in the human brain. **(A)** The number of brain eQTL with target genes lying more than 500 kbp and over-lapping with predicted chromatin interaction in seven brain cell types. **(B)** UCSC browser snapshot showing a Brain eQTL and its target gene *SOX10* connected with predicted long-range chromatin interaction in oligodendrocyte precursor (OPC).

Intersecting our predicted chromatin-interaction with available expression quantitative trait locus (eQTL) in the brain (Ng et al., 2017) using PGLtool (Greenwald et al., 2017) revealed possible cell-type in which the eQTLs are connected to their target genes. In the absence of availability of chromatin-interaction in brain cells, it is not trivial to retrieve information about possible cell-type for the action of the published brain eQTLs. The results of the intersection of eQTL data-set and predicted chromatin interaction in seven brain cell types are provided in Supplementary File 1. In the intersection result, we found many eQTLs whose target gene lied more than 500 kbp away and were supported by predicted long-range chromatin interaction by scEChIA. The number of eQTLs with the target gene lying more than 500 kbp away and supported by predicted interaction is shown in Figure 3A and Supplementary Figure 4. One such example of the long-range effect is eQTL (rs12165519) of SOX10 expression in the brain. Our analysis revealed that eQTL (rs12165519) overlaps a peak of open-chromatin profile (ATAC-seq) in the brain and could be connected to target SOX10 promoter through a long-range chromatin interaction in oligodendrocyte precursor cells (Figure 3B).

#### Coverage of Genome-Wide Association Studies Mutations and Cell-Type Specificity

**FIGURE 4:**
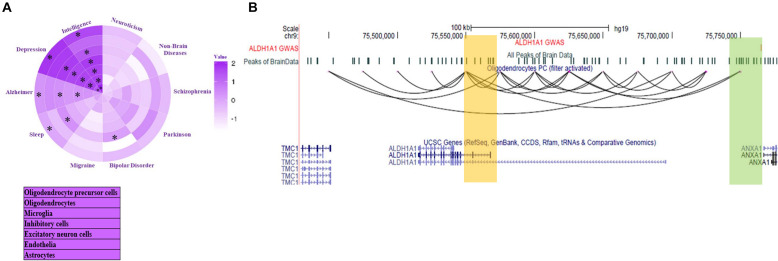
Inference about target genes for disease-associated mutations in Brain cells. **(A)** Enrichment of interaction among GWAS loci associated with mental disorder and gene promoters in seven brain cell-type. Star (*) represents *p* < 0.05. The *p*-value calculation was done using two proportion *z*-test. **(B)** The UCSC browser snapshot showing estimated chromatin interaction between the region with GWAS SNP (rs3758354) and promoter of ALDH1A1 gene in oligodendrocyte precursors (OPC).

Lake et al. (2018) investigated the enrichment of open chromatin signal within 100 kbp around GWAS mutations single nucleotide polymorphisms (SNPs) to estimate the cell-type specificity associated with a mental disorder. However, they did not try to find the target gene of GWAS SNP. Using our data-set of predicted chromatin-interaction in seven brain cell types, we found target genes of GWAS mutations associated with mental disorders. We label a gene as a target only when the 25 kbp genomic bin containing its promoter is interacting with the bin containing the GWAS mutation. We further compared the enrichment of mental disorders with GWAS loci overlapping with sites interacting directly with a gene. Enrichment was calculated by normalization with the fraction of GWAS SNP of non-brain disorder overlapping with sites interacting with promoters (promoter-connected). To find relative enrichment, we used a null-model comprising of GWAS mutations associated with non-brain disorders namely; Ulcerative colitis, lung cancer, breast cancer, bladder cancer, hepatitis A, hepatitis C, waist to hip, platelet count, bone mineral density, lung adenocarcinoma, and lung disease severity in cystic fibrosis. Compared to the null model, the higher enrichment of risk variants of a few mental disorders showed cell-type-specificity in connectivity to promoters, which corroborated with previous reports (Figure 4A). Such as, Alzheimer’s disease risk variants had higher enrichment in promoter-connected regions in microglia (Figure 4A and Supplementary Table 3). It has been reported that microglia signature genes have higher activity in the cortex on the development of late-onset Alzheimer’s disease (Zhang et al., 2013).

Our analysis also revealed a few genes with unknown associations with mental disorders. While for others, it revealed the possible cell type involved in disease development through the gene. Such as a region containing a mutation (SNP id: rs3758354) associated with schizophrenia and bipolar disorder and depression appear to be interacting with promoter of gene *ALDHA1* in oligodendrocyte precursor (Figure 4B). Interestingly *ALDHA1* is also known to be involved in the activation of retinoic acid receptor (RXR) for proper differentiation of oligodendrocyte precursors (Huang et al., 2011). However, its link with the SNP rs3758354 is not known, especially in oligodendrocyte precursor cells. More such results can be seen in Supplementary Figure 5 and Supplementary File 2. Many predicted target genes of GWAS mutations lay more than 500 kbp away (see Table in Supplementary File 2).

#### Targets of Human Accelerated Regions in Different Brain Cell Types

**FIGURE 5:**
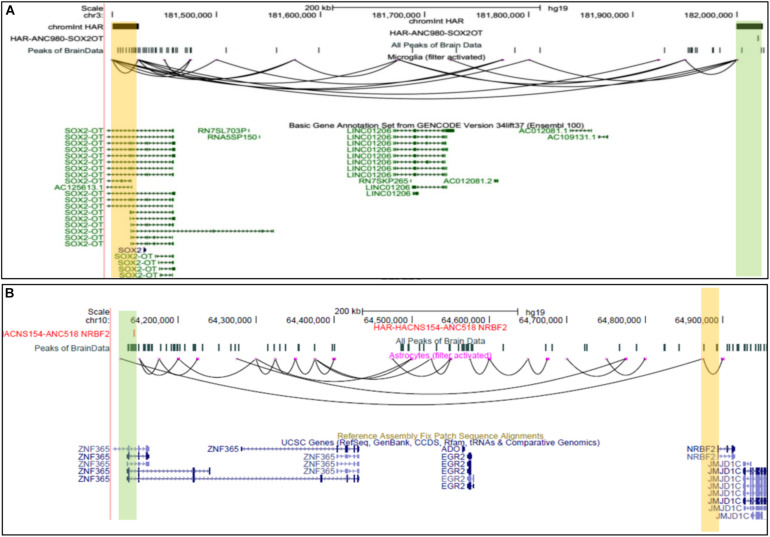
UCSC browser snapshots for genes connected to Human accelerated elements (HARS). **(A)** The UCSC browser snapshot, showing an interaction estimated to exist between a genomic bin containing a human-accelerated-region (HAR) and promoter of *SOX2OT*, in microglia. **(B)** The snapshot showing an interaction between a region with HAR and a promoter of *NRBF2* gene which is associated with Alzheimer’s disease.

Multiple Human accelerated regions (HARS) have been discovered; however, the mechanism of effect and influence is known only for a few HARS (Hubisz and Pollard, 2014). Given the fact that humans have more complex Brain structures than other species, our prediction could be a valuable resource to find target genes for HARS in brain cells. Hence, we intersected genomic sites involved in predicted chromatin-interaction with known HARS (Hubisz and Pollard, 2014). Our analysis revealed several target genes for HARS, provided in the Supplementary File 3 (see Figure 5). Such as scEChIA predicted interaction between a HAR named *ANC980* and promoter of gene *SOX2OT* (Figure 5A). *SOX2OT* is known to have multiple transcription-start sites (Amaral et al., 2009) and a role in the regulation of expression of *SOX2* and neurogenesis. Our result also revealed another interesting interaction between a HAR (ANC518) and promoter region of the *NRBF2* gene in astrocytes. The HAR ANC518 is located in the intron of gene *ZNF365* and appeared to be interacting with the promoter of *NRBF2* lying more than 500 kbp away (Figure 5B). Hence the prediction of such distal interaction (distance > 500 kbp) could not have been possible by current methods using a single-cell open-chromatin profile. *NRBF2* gene also seems to have detectable expression astrocytes (Supplementary Figure 5). *NRBF2* gene is known to be associated with Alzheimer’s disease, which some researchers have hypothesized to be a human-specific disorder (Finch and Austad, 2015).

### Insights About Regulatory Transcription Factors From Predicted Distal Chromatin Interactions

**FIGURE 6:**
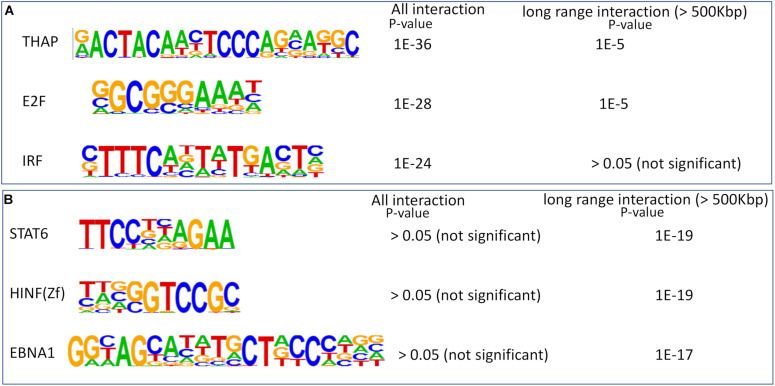
Enrichment of transcription factor motifs at sites with predicted chromatin-interaction in endothelial cells. **(A)** Top 3 enriched motifs at sites with all kinds of chromatin interaction (both short and long-range). The *p*-value of enrichment in two types of sites (all and only long-range) are also shown. **(B)** Top 3 enriched motifs at sites with long-range chromatin interactions. The *p*-values of enrichment in two types of sites are also shown. None of the top 3 enriched motifs in sites with predicted long-range interaction is enriched in loci with all predicted interactions.

In order to further elucidate the importance of detecting long-range chromatin contacts to infer regulatory networks in brain cells, we performed enrichment of transcription factor (TF) motif at non-promoter sites with predicted chromatin interactions. First, we performed motif enrichment analysis using HOMER (Heinz et al., 2010) for non-promoter genomic loci with chromatin interactions in endothelial cells. Then we selected non-promoter genomic loci with long-range chromatin interactions (>500 kbp) in endothelial cells. We found that most of the TF motifs enriched in all interacting sites also had significant enrichment in genomic loci with long-range interactions (Supplementary File 4). However, among top 3 enriched motifs in all genomic-loci with predicted chromatin contact, interferon regulatory factors (IRF) did not appear as enriched in sites with long-range interactions (>500 kbp) in endothelial cells (Figure 6A). A few other TF motifs highly enriched in genomic-loci with predicted long-range contact did not appear to have significant enrichment in sites with all chromatin interactions in endothelial cells. Such as top 3 TF motifs [*STAT6*, histone nuclear factor P (*HINFP*), and *EBNA1*] enriched in sites with long-range interactions in endothelial cells had no significant enrichment in sites with all kinds of chromatin interactions (Figure 6B). *EBNA1* is a viral protein associated with the Epstein-Barr virus. The role of HINFP (or MIZF) in endothelial cells need further investigation. However, the most interesting enriched motif is for TF STAT6, which get activated in endothelial cells from the brain due to external stimuli, as reported by few studies (Fasler-Kan et al., 2010; Tozawa et al., 2011; Dozio and Sanchez, 2017). Such a result suggests that STAT6 could be poising or controlling gene expression in endothelial cells through long-range chromatin interactions. It also highlights the fact that our method can create the possibility of making such insights about the regulatory action of TFs in cells using their scATAC-seq profile.

## Discussion

The problem of predicting chromatin interaction using single-cell epigenome profiles can be partially solved using co-accessibility. However, co-accessibility among genomics sites could be due to several reasons; therefore, previous methods were limited to predicting interaction within 500 kbp. Our approach breaks such barriers by using pre-existing knowledge as a prior for calculating the constrained estimate of chromatin-interaction. Our adaptive L1 normalization approach for estimating Gaussian graphical model and noise reduction through matrix factorization predicts a higher number of distal interactions (distance >500 kbp) using a single-cell open-chromatin profile than existing methods. We have also shown that our method could be better for few sparse single-cell open-chromatin profiles than existing similar methods, even in terms of accuracy.

Chromatin interaction prediction using a single-cell open chromatin profile can be useful in multiple ways. The predicted chromatin-interaction in seven brain cell types in this study could be a valuable resource for researchers to understand regulation in the human brain. Especially for cells in the natural state from the *in vivo* brain sample, the chromatin-interaction profile availability is rare. The utility of predicted long-range chromatin-interaction by scEChIA is reflected by the high number of overlapping brain eQTL and target gene contacts (for 1,000–4,500 eQTLs) with prediction. Using our tool and predictions, one can make multiple inferences such as: cell-type specificity of the target of GWAS loci, novel associations between genes and alternative promoters with diseases, targets of HARS and alternative splicing due to *cis*-regulation. Such as, our analysis reveals that one of the promoters of *SOX2OT* gene could be regulated by a HAR, and it could have a human-specific mechanism of controlling brain architecture and function. Our prediction of chromatin interaction in astrocytes revealed a connection between a HAR and *NRBF2* gene lying more than 500 kbp apart as a very relevant example. The autophagy associated gene *NRBF2* is known to have a reduction of expression in the human brain with Alzheimer’s disease (the seventh cause of death worldwide) (Lachance et al., 2019). Thus our method has the potential to highlight chromatin-interactions for making insight about clinically relevant regulatory mechanisms.

Previously other studies have highlighted a few examples of the regulatory effect of TFs by long-range chromatin interaction. A very relevant example is the priming of ESCs by NANOG (Novo et al., 2018). Novo et al. (2018) showed that long-range promoter-SE interactions are more prevalent in ESCs than in *Nanog*-deficient ESCs. Our result showing differential enrichment of TF motifs in sites with all predicted interactions and only long-range contacts in endothelial cells also highlights an interesting regulatory pattern. Both IRF and STAT6 are involved in the inflammatory response in endothelial cells (Tozawa et al., 2011; Yan et al., 2017). We found the IRF motif to be enriched at the site with chromatin interaction but missing at genomic loci with distal interactions. However, STAT6 motif was enriched only at sites with distal interactions in brain endothelial cells. Thus, our results generated a hypothesis that STAT6 could be preferably activating genes in brain endothelial cells through long-range chromatin contact, and IRF could be acting through short-range chromatin-interaction. Such examples highlight the utility of our method in inferring gene-regulatory networks using single-cell open chromatin profiles. Especially for less-abundant cells from *in vivo* samples, it could prove to be highly useful in inferring gene-regulatory networks influenced by long-range chromatin interactions.

## Data Availability Statement

The original contributions presented in the study are included in the article/Supplementary Material, further inquiries can be directed to the corresponding author.

## Author Contributions

VK and NP designed the project and wrote the manuscript. NP implemented the code for the method. SM helped in improving the code and to do some of the analysis. OC helped in analysis related to the GWAS and HARs. All authors contributed to the article and approved the submitted version.

## Conflict of Interest

The authors declare that the research was conducted in the absence of any commercial or financial relationships that could be construed as a potential conflict of interest.

## Publisher’s Note

All claims expressed in this article are solely those of the authors and do not necessarily represent those of their affiliated organizations, or those of the publisher, the editors and the reviewers. Any product that may be evaluated in this article, or claim that may be made by its manufacturer, is not guaranteed or endorsed by the publisher.

## Footnotes

1
https://www.4dnucleome.org/
