# Supplementary material for: Improving Chromatin-Interaction Prediction Using Single-Cell Open-Chromatin Profiles and Making Insight Into the Cis-Regulatory Landscape of the Human Brain
Source: Front Genet. 2021 Oct 8;12:738194. doi: 10.3389/fgene.2021.738194 (PMC8533004; doi:10.3389/fgene.2021.738194)
Supplement: Supplementary file 1 [file Data_Sheet_1.PDF]

Supplementary Material

Implementation details

Chromatin interaction prediction

We calculated the regularization parameter using known HiC profile not belonging to target cell-type whose scATAC-seq profile is used for chromatin-interaction prediction. For this purpose we used the equation (3) mentioned in main manuscript. The estimated regularisation parameter was passed as rho matrix in the graphical Lasso model using the function glassoFast. The function glassoFast is a speedy alternative to the Glasso function of Glasso R package (Friedman et al. 2008).

GWAS SNP enrichment calculation

We did GWAS based study to compare the enrichment of different mental illnesses with GWAS loci overlapping with sites interacting directly with a gene. Enrichment was calculated by normalization with the fraction of GWAS SNP of non-brain diseases overlapping with sites interacting with gene. The null model incorporating GWAS SNPs associated with non-brain diseases was used to find relative enrichment. Thus enrichment score for a brain disease in a cell-type is calculated as the ratio defined as:

$$ratio = \frac{fraction\ of\ a\ brain\ disease\ GWAS\ SNPs\ \ on\ sites\ interacting\ with\ gene}{fraction\ of\ non - brain\ GWAS\ SNPs\ on\ sites\ interacting\ with\ gene}$$

Two proportion z-test was applied to compare the two fractions (proportions). The corresponding Z-test was converted to P-value.

Motif enrichment analysis

We achieved motif enrichment analysis using HOMER, *i.e.,* Hypergeometric Optimization of Motif Enrichment. The size parameter is a fragment size that was used for motif finding and it was kept as size=200. The -nomotif parameter was used so as not to search for de novo motif enrichment. And the hg19 version was used as the genome.

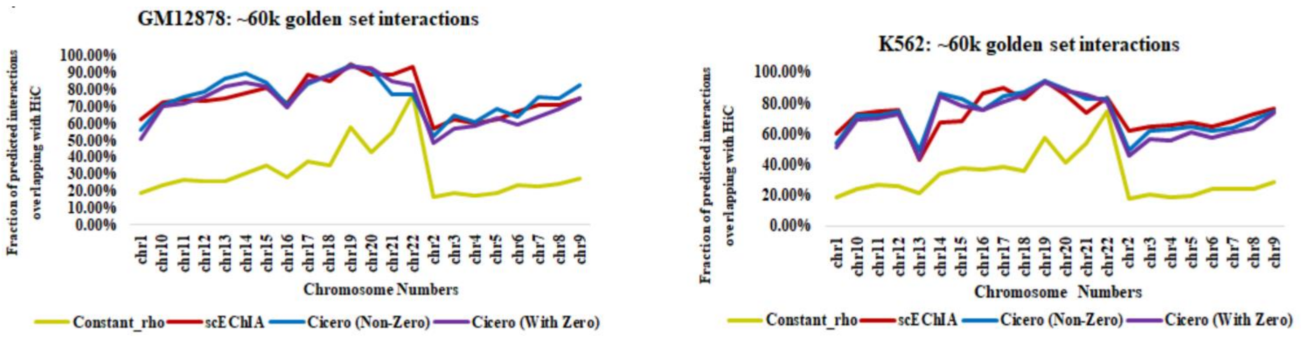

Supplementary Figure 1: Evaluation of the accuracy of prediction of chromatin interaction using scATAC-seq profile from two cell-types. The accuracy is measured as the fraction of all predicted chromatin interactions which overlap with enriched interactions in HiC profile. Here the number of enriched chromatin interactions from HiC profile was kept as 60000 for every chromosome.

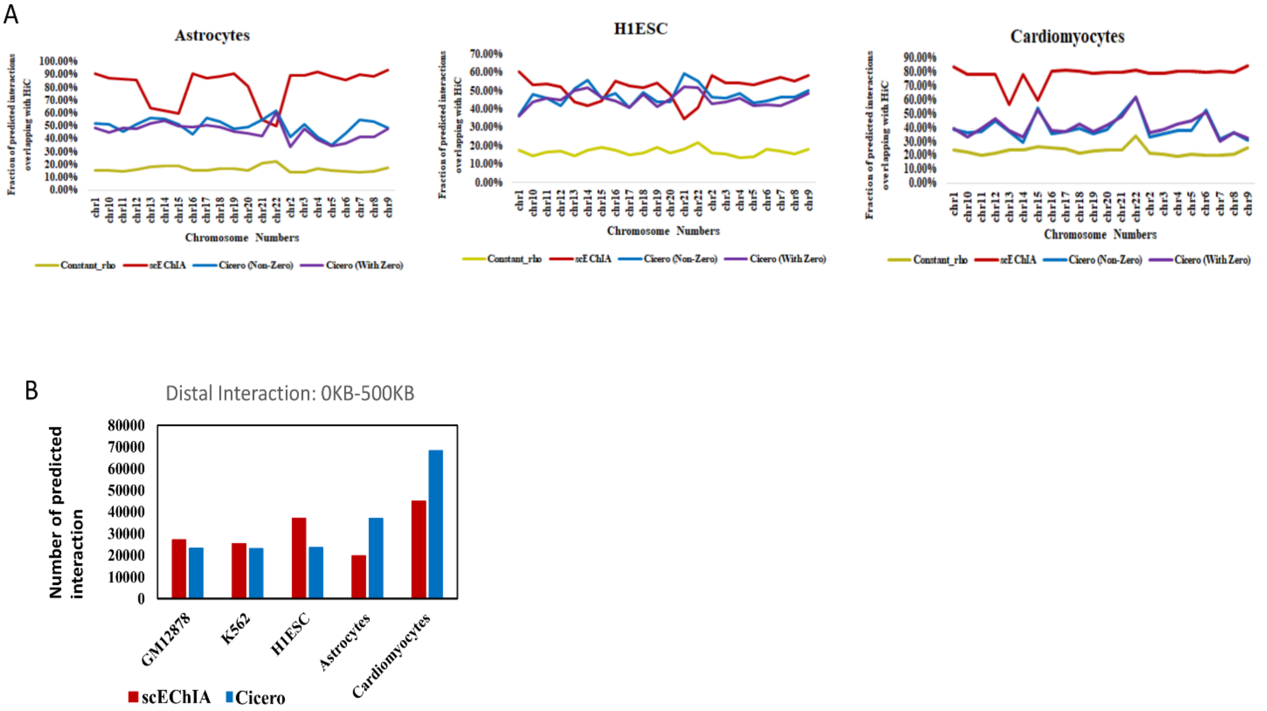

Supplementary Figure 2: Evaluation of predicted chromatin-interactions (A) Evaluation of the accuracy of predicting chromatin interaction using scATAC-seq profile from 3 cell-types, when the number of positive sets chosen from HiC is based on the sizes of chromosomes. The accuracy is measured as the fraction of all predicted chromatin-interactions which overlap with enriched. Interactions in HiC profile. (B) the number of predicted interactions within 500kb in different cell types by cicero and scEChIA.

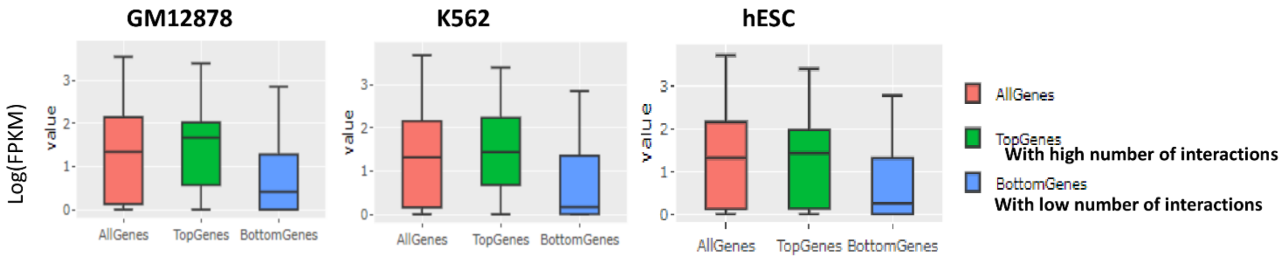

Supplementary Figure 3: Analysis of effect of predicted chromatin-interactions on gene-expression. Here the expression values of top 50 genes with highest and lowest number interactions at their promoter are shown for 3 cell types (GM12878, K562, hESC) as boxplot. Such as in panel labelled as GM12878, the expression values of top 50 genes which have high or low number chromatin interactions are shown.

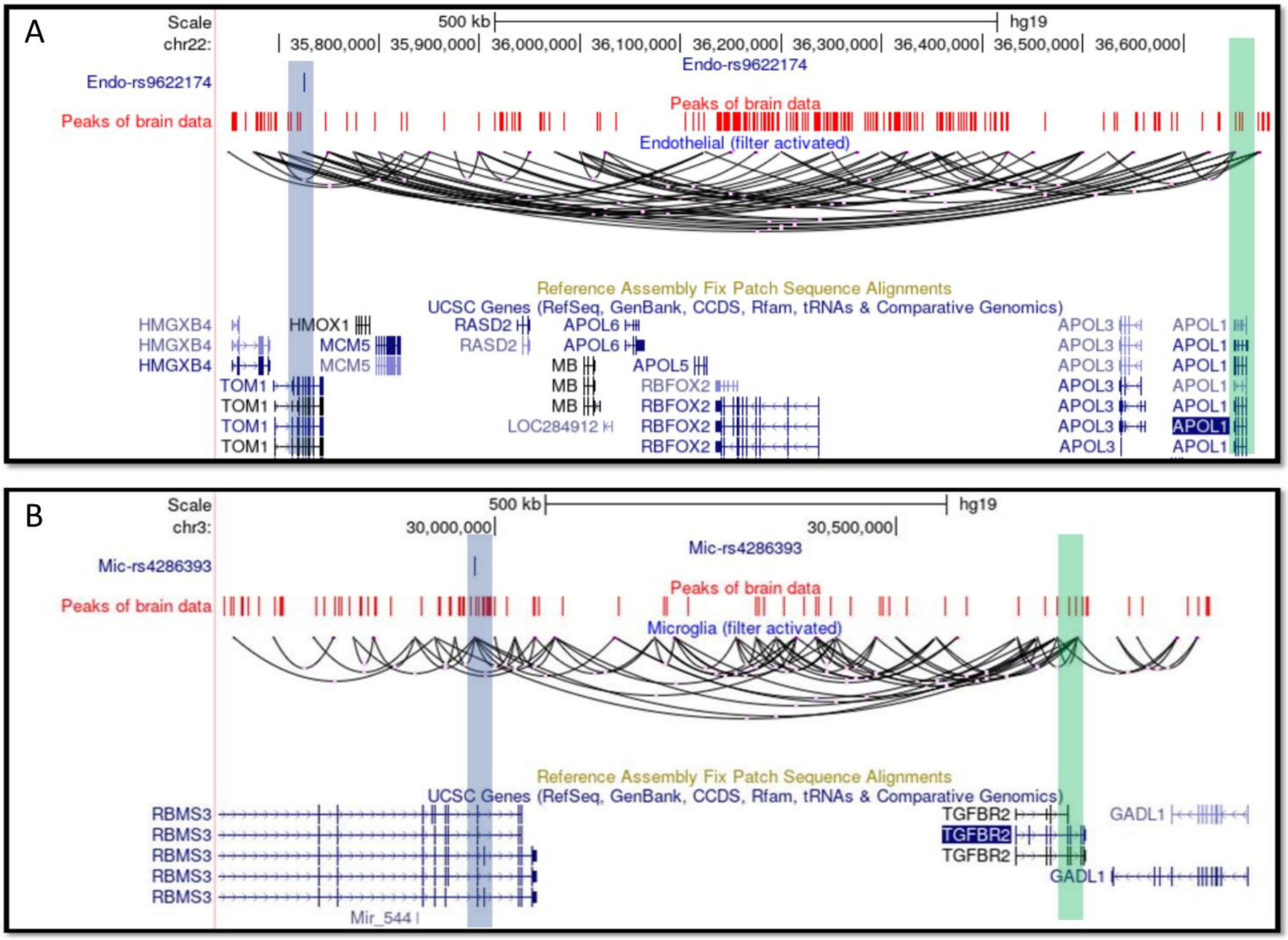

Supplementary Figure 4: UCSC track snapshot showing brain eQTL (expression quantitative trait locus) showing their connection with known target genes through predicted long-range chromatin interaction. (A) Snapshot of predicted chromatin interaction profile in endothelial cells where target

gene is shown connected APOL1 to its known eQTL in the brain. (B) it shows the connection of a brain QTL (rs4286393) with its known target gene lying more than 500 kbp.

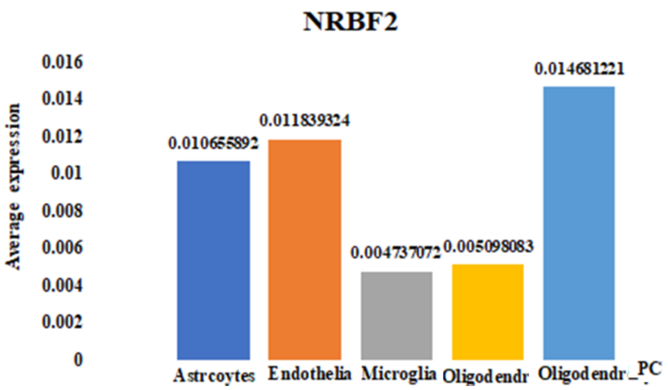

Supplementary Figure 5: Average expression of NRBF2 in brain cells. NRBF2 seems to have detectable expression in single-cells of Astrocytes in Song et al. scRNA-seq data-set.

Supplementary Table 1: Number of top enriched interactions (positive set) chosen from each chromosome according to their size to find the accuracy of scEChIA and other methods.

Chromosome	Size in BP	Number of Interaction
1	24,89,56,422	60000
2	24,21,93,529	58370
3	19,82,95,559	47790
4	19,02,14,555	45842
5	18,15,38,259	43751
6	17,08,05,979	41165
7	15,93,45,973	38403
8	14,51,38,636	34979
9	13,83,94,717	33353
10	13,37,97,422	32245
11	13,50,86,622	32556
12	13,32,75,309	32120
13	11,43,64,328	27562
14	10,70,43,718	25798
15	10,19,91,189	24580
16	9,03,38,345	21772
17	8,32,57,441	20065
18	8,03,73,285	19370
19	5,86,17,616	14127
20	6,44,44,167	15531
21	4,67,09,983	11257
22	5,08,18,468	12247

Supplementary Table 2 : Number of predicted chromatin interactions in seven cell-types using scATAC-seq profile published by Lake et al.

**Cell type**	**Number of predicted interactions**
Astrocytes	94843
Endothelia	65967
Excitatory Neuron	82784
Inhibitory	110874
Microglia	188857
Oligodendrocytes precursors	130234
Oligodendrocytes	25838

Supplementary table 3: The value of enrichment of interaction among GWAS loci associated with mental disorder and gene promoters in 7 brain cell-type.

Diseases	Astrocytes	Endothelial	Excitatory neuron cells	Inhibitory cells	Microglia	Oligo-dendrocytes	Oligodendrocyte Precursor cells
**Nonbrain Disease**	0.999	1.000	1	1	1.000	0.999	1
**Alzheimer**	1.204	1.191	1.308	1.197	1.390	1.151	1.325
**Intelligence**	1.569	1.435	1.622	1.461	1.594	1.333	1.599
**Migraine**	1.118	1.101	0.920	0.945	1.207	0.703	1.093
**Neuroticism**	0.984	0.887	1.092	1.028	1.191	0.827	1.159
**Parkinson**	1.060	1.1458	1.215	1.178	1.153	1.145	1.163
**Schizophrenia**	1.0367	1.0452	1.235	1.044	1.161	1.028	1.182
**Sleep**	1.1729	1.0369	1.149	1.124	1.394	0.886	1.26
**Depression**	1.442	1.438	1.623	1.435	1.678	1.537	1.652
**Bipolar Disorder**	1.0962	1.0294	1.120	1.083	1.334	0.529	1.176

Supplemental file -1: It contains the intersection between published eQTL and predicted chromatin interactions in seven brain cell-types.

Location: http://reggen.iiitd.edu.in:1207/scEChIA/supplementary/supplemental-file-1.zip

Supplemental file-2 : It contains the predicted target gene-promoters of GWAS mutations found using predicted chromatin interactions in seven brain cell-types.

Location: http://reggen.iiitd.edu.in:1207/scEChIA/supplementary/supplemental-file-2.zip

Supplemental file-3 : This file contains the information of chromatin interaction between regions containing HARS and promoter of genes.

Location: http://reggen.iiitd.edu.in:1207/scEChIA/supplementary/supplemental-file-3.zip

Supplemental file-4: The result of motif enrichment analysis for two kinds of non-promoter genomic loci: 1) loci with all predicted chromatin-interaction in Brain endothelial cells 2) loci with only predicted long-range chromatin interactions in Brain endothelial cells.

Location: http://reggen.iiitd.edu.in:1207/scEChIA/supplementary/supplemental-file-4.zip
